# Potentially heritable biomarkers of neurodegeneration in a marmoset model of Alzheimer’s disease

**DOI:** 10.1002/alz.090952

**Published:** 2025-01-03

**Authors:** Sonal Kumar, Catrina Spruce, Annat Haber, Laura Schultz, Hasi Huhe, Takeshi Murai, Sang‐Ho Choi, Stephanie Hachem, Yongshan Mou, Seung‐Kwon Ha, Jung Eun Park, Lauren Bailey, Lauren Mongeau, Andrew DeSana, Brianne Stein, Lauren K Hayrynen Schaeffer, Gregg E Homanics, Afonso C Silva, Stacey J Sukoff Rizzo, Gregory W Carter

**Affiliations:** ^1^ The Jackson Laboratory, Bar Harbor, ME USA; ^2^ Tufts University Graduate School of Biomedical Sciences, Boston, MA USA; ^3^ The Jackson Laboratory for Genomic Medicine, Farmington, CT USA; ^4^ University of Pittsburgh School of Medicine, Pittsburgh, PA USA

## Abstract

**Background:**

Alzheimer’s disease (AD) is the most common global cause of dementia, with no real cure available. Despite extensive genetic findings from large‐scale genetic associations and similar studies, our understanding of AD is largely hindered by its long, asymptomatic progression with limited access to human brain tissue. Animal models like the marmoset allow for longitudinal analysis of disease by enabling the assaying of disease‐specific phenotypes that mimic human pathophysiology. Here, we have analyzed genetic associations with disease biomarkers in a cohort of wild type and *PSEN1* C410Y mutant marmosets.

**Method:**

Whole genome sequencing was carried out for a cohort of largely wild‐type and a few *PSEN1* mutant carriers. A wide variety of disease associated biomarkers were analyzed to assess for the progression of neurodegeneration. The correlation structure underlying collected biomarkers and related phenotypes was calculated, separating for sex. Composite phenotypes were computed based on correlation strength, and genetic associations studied via QTLs for composite and individual phenotypes.

**Result:**

Females showed greater correlations between disease specific biomarkers like total tau (tTau), neurofilament light protein (NfL), the astrocytic marker GFAP, and β‐amyloid peptides with 40 and 42 amino acids, as well as the Aβ42:Aβ40 ratio. Correlations between markers were found to be weaker but still significant in males. Variants predicted to be pathogenic were found for many genetic loci implicated by human genome‐wide association studies. Standing genetic variation in the marmosets was found akin to what is seen in humans, but overall population structures varied significantly between the two species.

**Conclusion:**

Correlations between AD‐relevant biomarkers in marmosets exceeded those observed in humans, which were not explained by age. This suggests a strong potential genetic contribution. Leveraging possible heritable associations with disease markers discovered in the marmosets to explain the progression of neurodegeneration could directly provide insights into human AD biology from a genetic point of view.

